# How Laboratory Innovations Are Shaping the Future of Multiple Myeloma Care

**DOI:** 10.3390/cancers18081275

**Published:** 2026-04-17

**Authors:** Joana Caetano, Ana Marta Pires, Carlos Costa, Rui Bergantim, Adriana Roque, Patrícia Ferraz, Maria Rosário Cunha, Niccolo Bolli, Noemi Puig, Cristina João

**Affiliations:** 1 Hemato-Oncology Unit, Fundação Champalimaud, 1400-038 Lisbon, Portugal; joana.caetano@research.fchampalimaud.org; 2 NOVA Medical School, NOVA University of Lisbon, 1169-056 Lisbon, Portugal; 3 Clinical Pathology Department, Unidade Local de Saúde Trás-os-Montes e Alto Douro, 5000-508 Vila Real, Portugal; amcpires@chtmad.min-saude.pt; 4 Hemato-Oncology Unit, CUF Hospital Cascais, 2750-663 Cascais, Portugal; carlos.bruno.costa@jmellosaude.pt; 5 Hematology Department, Unidade Local de Saúde São João, 4200-319 Porto, Portugal; rui.bergantim@chsj.min-saude.pt; 6 Cancer Drug Resistance Group, Institute of Molecular Pathology and Immunology (IPATIMUP), Universidade do Porto, 4200-135 Porto, Portugal; 7 i3S-Institute for Research and Innovation in Health, Universidade do Porto, 4200-135 Porto, Portugal; 8 Clinical Hematology Department, Unidade Local de Saúde de Coimbra, 3004-561 Coimbra, Portugal; 9 Physiology Institute, Faculdade de Medicina, Universidade de Coimbra, 1649-023 Coimbra, Portugal; 10 Hematology Department, Unidade Local de Saúde Trás-os-Montes e Alto Douro, 5000-508 Vila Real, Portugal; pferraz@chtmad.min-saude.pt; 11 Clinical Pathology Department, Unidade Local de Saúde de Coimbra, 3004-561 Coimbra, Portugal; rosariocunha@chuc.min-saude.pt; 12 Hematology Unit, Foundation IRCCS Ca’ Granda Ospedale Maggiore Policlinico, 20122 Milan, Italy; niccolo.bolli@unimi.it; 13 Department of Oncology and Hemato-Oncology, University of Milan, 20122 Milan, Italy; 14 Hematology Department, Hospital Universitario de Salamanca, Instituto de Investigación Biomédica de Salamanca (IBSAL) y Instituto de Biología Molecular y Celular del Cáncer (IBMCC), Universidad de Salamanca, Consejo Superior de Investigaciones Científicas (USAL-CSIC), CIBERONC, 37007 Salamanca, Spain; noemipuig@usal.es

**Keywords:** multiple myeloma, diagnosis, prognosis, measurable residual disease, mass spectrometry, circulating tumor cells, cell-free DNA, artificial intelligence

## Abstract

Multiple myeloma is a complex hematologic malignancy characterized by significant biological heterogeneity, a relapsing–remission clinical course, and a continuously evolving therapeutic landscape. Accurate and timely laboratory assessment is central to disease management, supporting diagnosis, risk stratification, evaluation of treatment response, and long-term monitoring. Despite major advances in therapy, a critical need remains for laboratory tools that can detect disease with greater sensitivity, capture spatial and clonal tumor heterogeneity, and reflect the true depth of treatment response beyond conventional serological and bone marrow-based criteria. Recent laboratory innovations have the potential to transform myeloma care by enabling earlier detection, more accurate prognostication, and personalized therapeutic strategies. This review focuses specifically on innovative laboratory technologies for the diagnosis of multiple myeloma and the evaluation of treatment response. Within this scope, we examine the current diagnostic approaches and the role of high-throughput technologies for measurable residual disease assessment. We explore the emerging role of liquid biopsy approaches, including circulating tumor cells, cell-free DNA/RNA, and mass spectrometry for ultrasensitive detection of monoclonal proteins. We further discuss novel molecular biomarkers and the integration of artificial intelligence and machine learning tools to enhance data interpretation. The innovations reviewed here represent a shift in the contribution of laboratory medicine to myeloma care, offering a more precise, less invasive, and biologically informative framework for targeted and adaptive clinical decisions.

## Simple Summary

Multiple myeloma is a type of blood cancer that can be difficult to detect and monitor over time. This review examines how recent laboratory innovations are improving the way the disease is detected, followed, and treated. New technologies, such as advanced genetic testing, minimally invasive tests that analyze tumor-derived material in blood, and automated data analysis allow doctors to identify the disease earlier and monitor it more accurately during and after treatment. These tools also help to classify patients into different risk groups and support more personalized treatment decisions based on the characteristics of each patient’s disease. In addition, the integration of artificial intelligence is helping clinicians to interpret large amounts of clinical and biological data more efficiently. Overall, these laboratory advances are changing patient care by supporting earlier diagnosis, more precise monitoring, and more individualized treatment strategies, with the goal of improving outcomes and long-term disease management.

## 1. Current Laboratory Methods in Multiple Myeloma: An Overview

### 1.1. Established Diagnostic Approaches

Multiple myeloma (MM) is a complex hematological malignancy defined by the aberrant proliferation and accumulation of neoplastic plasma cells (PCs) within the bone marrow (BM). Rather than a singular disease entity, it should be understood as a clinical spectrum, involving a stepwise progression from monoclonal gammopathy of undetermined significance (MGUS), through asymptomatic smoldering MM (SMM), to symptomatic, active MM [1]. Historically, diagnosis relied mostly on clinical presentation, including the presence of CRAB features (Hypercalcemia, Renal insufficiency, Anemia, and Bone lesions). The need for earlier identification led to the incorporation of biomarker-driven criteria [2]. The Revised International Myeloma Working Group (IMWG) introduced Myeloma-Defining Events (MDEs), integrating specific biomarkers into the SLiM-CRAB framework to enable earlier and more accurate diagnosis (Table 1) [3]. These updated criteria represent a paradigm shift, enabling diagnosis and initiation of therapy before established end-organ damage occurs and emphasizing the crucial need for highly sensitive monitoring assays [4]. Although the precise etiology of MM remains unknown, numerous genetic abnormalities have been implicated in its pathogenesis [5,6]. MM is classically subdivided into mutually exclusive cases with rearrangements involving the immunoglobulin heavy (IGH) locus and cases showing hyperdiploidy, as outlined in the latest World Health Organization and International Consensus Classification ICC, while gene mutations are often regarded as later events [7,8]. Current clinical staging systems, such as the International Staging System (ISS), Revised ISS (R-ISS), and 2nd Revised ISS (R2-ISS), mainly use clinical measures (serum β2-microglobulin, serum albumin, lactate dehydrogenase, and cytogenetic alterations) [9,10,11]. The inherent complexity of MM, characterized by significant intra-tumoral heterogeneity and ongoing clonal evolution, limits traditional approaches as they often overlook the molecular and cellular factors influencing prognosis and response to therapy [12,13,14]. To address this issue, the International Myeloma Society and IMWG proposed the Consensus Genomic Staging, which defines high-risk cases based on specific genomic abnormalities. These include del(17p) (>20% clonal fraction) and/or *TP53* mutation; IGH translocations (t(4;14), t(14;16), t(14;20)) combined with 1q gain and/or del(1p32); monoallelic or biallelic del(1p32) with 1q gain; or elevated β2-microglobulin (≥5.5 mg/L) in patients with normal kidney function [15,16,17]. This refined approach integrates molecular and clinical data to improve risk stratification and guide therapeutic decision-making [18].

### 1.2. State-of-the-Art Response Evaluation

The advent of novel therapies achieving deeper and more durable responses has fundamentally redefined treatment goals in MM. Assessment of treatment response has evolved significantly beyond conventional complete response (CR) and stringent CR (sCR) criteria, now achieved by an increasing proportion of patients with current induction regimens and therefore, considered insufficient to establish prognosis. Instead, measurable residual disease (MRD) assessment has emerged as a powerful prognostic indicator to evaluate therapeutic efficacy [19,20,21]. MRD negativity is consistently associated with improved progression-free survival (PFS), whereas persistence of MRD following therapy confers a higher risk of relapse [22,23]. Two large meta-analyses established the strong prognostic value of MRD negativity and its association with PFS and overall survival (OS) in various disease and treatment settings, with even greater benefits in PFS for patients with MRD negativity at lower levels of sensitivity (10^−6^) [24,25]. Consequently, MRD status has been established as a relevant surrogate marker for PFS and potentially OS. Reflecting this evidence, on 12 April 2024, the Food and Drug Administration (FDA) unanimously approved the use of MRD as an early endpoint to support accelerated approval in MM clinical trials [26]. Recently, artificial intelligence-assisted serial MRD analysis has further refined risk stratification and PFS prediction [27]. MRD-guided approaches are anticipated to become standard practice and a critical tool for personalized disease management [28], and therefore, multiple clinical trials are evaluating strategies to individualize therapy in MM (Table 2).

In transplant-eligible newly diagnosed MM (NDMM), PERSEUS showed significantly higher and more sustained MRD negativity with daratumumab, bortezomib, lenalidomide, and dexamethasone (Dara-VRd) followed by autologous stem cell transplant (ASCT) and daratumumab plus lenalidomide maintenance than VRd alone (75.2% versus 47.5%; *p* < 0.001; 64.8% versus 29.7%, respectively), enabling treatment discontinuation after ≥24 months in selected patients [29]. The ongoing DRAMMATIC trial is evaluating MRD-adapted maintenance duration using annual MRD assessments [30]. The MASTER trial further established the feasibility of MRD-guided therapy cessation. After daratumumab, carfilzomib, lenalidomide, and dexamethasone (Dara-KRd) induction followed by ASCT and 2 phases of Dara-KRd consolidation in standard-risk patients, patients with sustained MRD negativity (<10^−5^) entered treatment-free observation (MRD-SURE phase). Progression risk after treatment cessation was 9% in standard-risk patients but increased to 47% in those with two or more high-risk cytogenetic abnormalities [31]. MASTER-2 is extending this approach after Dara-VRd and ASCT, including evaluation of ASCT deferral in MRD-negative patients and intensified therapy for MRD-positive patients [32]. MIDAS and MRD2STOP further supported MRD-guided consolidation and discontinuation strategies, showing improved outcomes for patients achieving deep MRD responses, including thresholds < 10^−7^ [33,34]. Higher prognostic value of longitudinal MRD dynamics rather than a single time-point assessment was indicated by FORTE, MAIA, ALCYONE, POLLUX, and CASTOR trials [35,36]. Belantamab mafodotin-based regimens (DREAMM-7 and DREAMM-8) reported deeper and more durable MRD negativity with prolonged PFS [37,38]. Additional studies (TOURMALINE-MM3/-MM4 and AURIGA) reinforced the clinical value of deep and sustained MRD negativity, particularly with longitudinal monitoring [39,40,41].

Evaluation of MRD requires highly sensitive and standardized methods that surpass conventional techniques, notably Next Generation Flow (NGF) and Next Generation Sequencing (NGS), both meeting the IMWG-recommended sensitivity of 10^−5^ [21]. Several clinical trials have incorporated both methods for MRD assessment, and a threshold of 10^−6^ has demonstrated greater clinical value, increasingly being considered the preferred target for more accurately defining MRD negativity [42,43,44,45,46,47,48].

NGF, developed, optimized, and validated by the EuroFlow consortium, uses an 8-color, 2-tube antibody panel integrating the assessment of 10 markers (CD38, CD138, CD45, CD19, CD27, CD56, CD81, CD117, cytoplasmic Igκ, and cytoplasmic Igλ). The selected markers allow reliable discrimination between normal and clonal PCs, as well as provide additional information on other cell populations, including those that allow hemodilution identification, a factor that can impact MRD accuracy [49]. Together with a novel sample-processing protocol that enables the analysis of a high number of cells (≥10^7^ nucleated BM cells), this approach reaches a sensitivity of 2 × 10^−6^ with thresholds for the lower limit of detection (LLOD) of 20 cells and the lower limit of quantification (LLOQ) of 50 cells [50]. Beyond NGF, alternative flow cytometry strategies have been developed to assess MRD using a single 10-color or 12-color tube [51,52,53]. In particular, the approach developed at Memorial Sloan Kettering Cancer Center demonstrated a comparable analytical sensitivity to the EuroFlow-based method, offering an alternative to laboratories lacking full NGF infrastructure. While these assays do not achieve the same degree of inter-laboratory standardization as NGF and are not formally recognized by the IMWG for MRD reporting, if rigorously validated, they represent a practical and scalable alternative. NGS identifies and tracks tumor-specific immunoglobulin V(D)J rearrangements of the immunoglobulin heavy chain (*IGH*) variable region to define clonality in PCs. ClonoSEQ (Adaptive Biotechnologies, Seattle, WA, USA), which uses the rearrangement of the immunoglobulin genes (*IGH*, *IGK*, and *IGL*), is currently the only FDA-approved MRD assay [54]. Other alternative commercial platforms and in-house protocols have also been developed [55,56]. Both methodologies are broadly applicable to most patients. NGS offers the advantage of allowing deoxyribonucleic acid (DNA) storage, avoiding the need for fresh samples. However, not all patients have V(D)J rearrangements suitable for tracking. It requires a lower number of cells (approximately 3 million for an input of 20 μg of DNA). Conversely, NGF requires a higher number of cells and a fresh BM sample. It offers faster processing and turnaround time, and intrinsic quality control, allowing the detection of sample hemodilution and specimen representativeness. Despite their high sensitivity, both NGF- and NGS-based MRD assessments are limited by their reliance on BM aspirates, as they typically contain the greatest disease burden. However, BM involvement can be spatially heterogeneous, and a single draw may fail to capture focal areas of disease. Technical factors, such as hemodiluted BM samples, operator variability, and low cellularity, can affect MRD assessment accuracy and practicality. Furthermore, BM sampling is an invasive procedure that can cause patient discomfort and pose logistical challenges [57].

**Figure 1:**
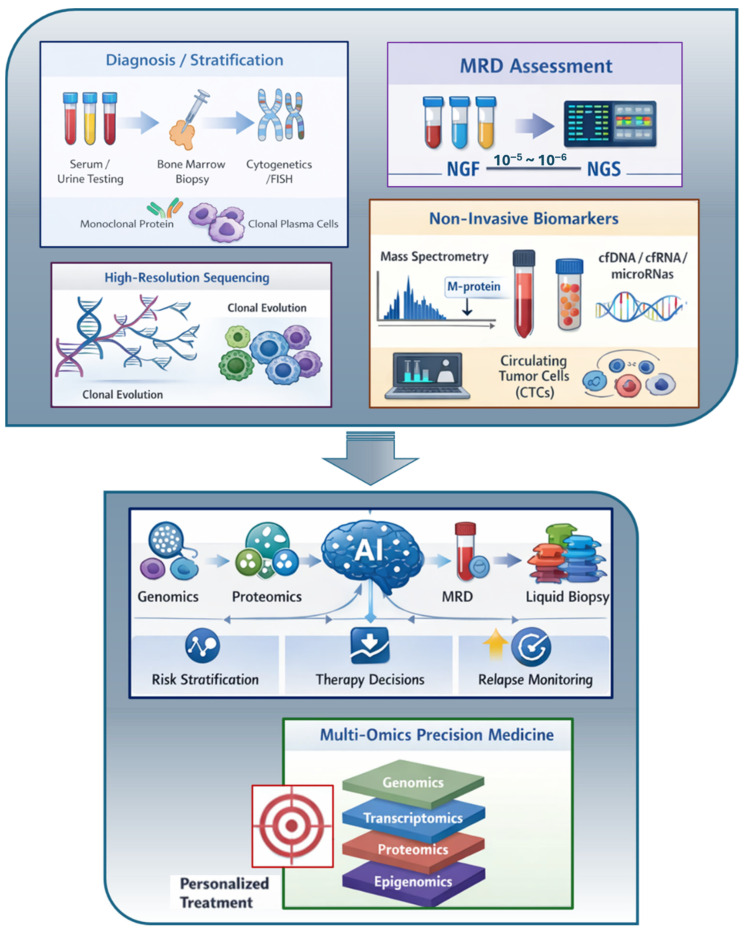
Graphical overview of laboratory innovations in MM from established diagnostic criteria (top box—blue) and MRD evaluation (purple) to high-resolution sequencing (grey), and non-invasive biomarkers (orange). Arrows indicate workflow progression and data integration (bottom box-white) converging into AI-integrated multi-omics approaches to support precision medicine (green).

Future progress in MM management depends on introducing innovative, highly sensitive, minimally invasive technologies to refine diagnosis, risk stratification, and treatment monitoring and tailoring. Multiple innovative methodologies are under investigation for possible integration in clinical practice (Figure 1).

## 2. Emerging Non-Invasive Insights into MM

BM aspirations remain the gold standard for diagnosis and MRD assessment. However, BM sampling is limited by the patchy distribution of MM, and because neoplastic PCs are unevenly distributed, a single-site aspirate may not reflect overall infiltration. Blood-based methodologies offer advantages such as reduced invasiveness, greater accessibility, and the potential for ongoing monitoring. They are complementary rather than interchangeable, and may help overcome limitations such as spatial heterogeneity and bias [58]. In the following sections, some of the most promising emerging non-invasive laboratory approaches will be discussed.

### 2.1. Mass Spectrometry (MS)

The unprecedented depth of response achieved with novel therapeutic strategies in MM has reshaped disease monitoring by exposing the limitations of conventional laboratory techniques. While many patients now achieve complete response by standard criteria, residual disease frequently persists below the detection limits of routine assays, ultimately driving disease recurrence [59]. This shift has moved the clinical focus away from initial disease detection toward the need for highly sensitive tools capable of identifying and tracking MRD, ideally through non-invasive methods that allow longitudinal assessment during prolonged disease control [60,61,62,63].

Serum protein electrophoresis (SPE), immunofixation electrophoresis (IFE), and serum free light chain (sFLC) assays remain widely available, cost-effective, and adequate for the diagnosis of MM in most patients. These techniques are still essential in clinical practice, particularly where specialized assays are unavailable or conservative treatment is needed. In these scenarios, conventional methods continue to provide robust and actionable information, allowing early detection of biochemical changes before overt clinical relapse. However, their limited analytical sensitivity and potential analytical interferences, including those arising from therapeutic monoclonal antibodies, restrict their utility in patients who achieve deep responses early in therapy. The limitation is not diagnostic capability per se, but the inability of conventional assays to detect very low levels of residual monoclonal protein (MP) in the context of highly effective treatments [60,61,62,63,64]. This limitation is particularly apparent in cases of oligo-secretory and traditionally non-measurable disease. Patients in these groups have been underserved by standard serological monitoring methods, which lack sensitivity. MS, however, allows for serum-based monitoring at levels far below conventional thresholds, enabling assessment that previously depended solely on BM assessment or imaging alone [65]. In MM patients with renal impairment, MS performance remains limited and requires further validation in this setting.

MS has emerged as a promising alternative to conventional techniques, enabling highly sensitive and specific detection of monoclonal immunoglobulins in serum and supporting residual disease assessment in peripheral blood. Rather than being universally implemented as a replacement for serum protein electrophoresis or immunofixation, MS is currently being integrated into tiered or network-based laboratory strategies that reflect the practical realities of MRD evaluation. The most sensitive BM-based MRD techniques, although analytically powerful, remain technically demanding, logistically complex, and not universally accessible. From a laboratory perspective, however, the long-term objective may involve the gradual full replacement of conventional electrophoretic and immunofixation methods, a direction already demonstrated in major centers such as the Mayo Clinic, where MALDI-TOF MS (Mass-Fix) has been validated and implemented as a high-throughput alternative [66]. By providing a sensitive, non-invasive serum method suitable for repeated assessments, MS may support clinical workflows without requiring immediate universal adoption and aligns with the need for practical, scalable approaches to MRD monitoring [67].

Beyond improved analytical sensitivity, MS-based assays can also capture additional molecular features of monoclonal proteins, including post-translational modifications such as light chain N-glycosylation, providing biological information that is not accessible through conventional electrophoretic techniques and may carry independent clinical relevance [68,69]. Emerging data also suggest that MS-based detection of light chain N-glycosylation may refine risk assessment in precursor conditions. Early work by Mills et al. showed that mass spectrometry can directly detect glycosylated monoclonal light chains in serum, uncovering molecular heterogeneity that may affect clearance, aggregation, and tissue deposition. This established proof-of-concept that MS can characterize qualitative features of monoclonal proteins beyond simple quantification [70]. More recent work has expanded the clinical relevance of qualitative MS readouts by showing that monoclonal protein heterogeneity can evolve over time and under therapeutic pressure. In this context, Landazuri et al. demonstrated that longitudinal MS monitoring reveals qualitative changes in monoclonal protein composition—independent of concentration—that are not captured by electrophoretic techniques, supporting the role of MS as a tool for both quantitative and qualitative disease assessment [71].

In parallel, MS-based workflows have also addressed a clinically important challenge: distinguishing endogenous monoclonal proteins from therapeutic monoclonal or bispecific antibodies, such as daratumumab, teclistamab and others. Preliminary real-world data, including findings presented as a poster at the 2025 Annual Meeting of the Portuguese Society of Hematology, have shown that serum-based MS can reliably detect Teclistamab using antibody-specific mass signatures [72]. These early observations reveal inter-individual variability in teclistamab detection across patients, the underlying determinants of which remain to be established. This finding highlights an area requiring further investigation, raising the hypothesis that MS could, in the future, contribute to understanding treatment kinetics. Pello et al. systematically characterized the mass-to-charge (*m*/*z*) signatures of commonly used therapeutic antibodies and demonstrated that MALDI-TOF-based quantitative immunoprecipitation (QIP) MS (EXENT^®^) can reliably distinguish drug-related signals from residual disease [73].

Beyond these analytical advantages, interpretation of MS findings in patients receiving antibody-based therapies must also consider treatment-related biological and immunological factors, including immunoglobulin suppression, oligoclonal reconstitution, and the pharmacokinetic behavior of modern monoclonal and bispecific antibodies. These aspects highlight that longitudinal MS assessment requires close clinical–laboratory collaboration to ensure accurate contextualization of results during different treatment phases [74].

Reevaluating response assessment in the era of mass spectrometry has also highlighted limitations inherent to conventional CR definitions. According to IMWG criteria, CR is defined by negative serum and urine immunofixation, fewer than 5% PCs by BM morphology, and resolution of plasmacytomas [21,60]. However, urine collection and BM examination are frequently impractical in routine clinical practice, while the clinical value of isolated morphological assessment in patients achieving deep serological responses remains limited. Recent analysis in transplant-eligible cohorts suggests that neither BM PCs counting nor serum immunofixation status reliably discriminates PFS, whereas serum MS provides improved prognostic stratification, including among patients classified as “suspected CR” based on negative serum immunofixation [59]. These observations are consistent with prior evidence demonstrating that classical IMWG response categories, including CR and sCR, lose prognostic discrimination in the context of modern intensive therapies when compared with MRD-based assessment [63]. Multiple clinical studies have demonstrated that QIP-MS is more sensitive than immunofixation for detecting residual disease during treatment and follow-up, and that MS positivity among immunofixation-negative patients identifies subgroups with significantly different PFS, supporting its prognostic relevance [59,60,61,62].

Together, these observations support the role of MS as a serological tool to refine response assessment and suggest that BM aspiration in suspected CR may add limited value unless performed in the context of MRD testing.

#### Challenges to Clinical Implementation

Studies have demonstrated that persistent MS positivity is associated with inferior PFS, while sustained MS negativity over time appears to define patient subsets with particularly favorable outcomes. These findings support the concept of serum-based MRD, referring to the detection of residual MP using highly sensitive assays, as a clinically meaningful biomarker. These findings suggest that MS may play an important role in refining response assessment beyond the definition of CR, particularly when integrated with other MRD modalities [62,64]. However, MS-based monitoring in MM still faces several practical and biological limitations that need to be addressed before widespread adoption. These challenges are not primarily related to analytical standardization or inter-laboratory harmonization, which is substantially facilitated using fully automated platforms, but rather to the current scope of detectable analytes and the biological interpretation of certain MS findings. Present MS workflows do not yet include the detection of IgD or IgE isotypes and are still evolving toward fully validated assays for free light chains. In addition, MS relies exclusively on serum as the analytical matrix, which may limit its application in clinical settings where MP are more appropriately evaluated in alternative matrices, such as urine and tissue, including AL amyloidosis, until alternative matrices are available. In extramedullary (EM) disease, MS alone might be insufficient to confirm response, requiring complementary imaging. Importantly, the clinical significance of some low-level or atypical MS signals remains to be defined, underscoring the need for further work to establish robust reporting criteria and clinically meaningful response thresholds [75,76,77]. In addition, the availability of MS is currently concentrated in specialized centers, which may influence its integration into routine clinical workflows.

International recommendations increasingly support the integration of MS into laboratory workflows for plasma cell disorders. European consensus guidelines now endorse MS as a preferred method for detecting and characterizing monoclonal proteins in several clinical settings, particularly in the follow-up of patients achieving deep responses [60]. Complementary expert reviews also highlight MS as a likely successor to immunofixation as analytical performance and scalability continue to improve [67].

### 2.2. Circulating Tumor Cells (CTC)

Under physiological conditions, long-lived BM PCs do not enter the peripheral circulation, but in individuals with monoclonal gammopathies, neoplastic PCs can leave the BM into the peripheral blood (PB) [78]. Seminal work by Paiva and colleagues demonstrated that CTC are not merely a passive spillover from the marrow but represent a biologically and phenotypically distinct tumor compartment with prognostic relevance [79]. CTC released into the PB from the primary tumor site have been explored as a more comprehensive representation of overall disease burden. In specific disease subtypes, the requirement for highly sensitive detection approaches is even greater. Bone marrow-independent laboratory techniques able to capture systemic dissemination and clonal diversity beyond a single-site sample are particularly relevant in EM disease. Also, in oligosecretory MM and traditionally non-measurable disease, serology-independent approaches such as CTC enumeration that do not rely on MP secretion are needed when conventional methods are uninformative. In the context of MM complicated by renal impairment, as CTC detection does not depend on renal clearance of analytes and is unaffected by renal dysfunction, this strategy could be applicable, although no dedicated studies are available.

The frequency of PB involvement is largely dependent on the sensitivity of the methods used. While detection of CTC in NDMM patients using conventional morphological methods is limited, multiparametric flow cytometry (MFC) substantially increased sensitivity, allowing CTC identification in 70–87% of NDMM patients [80]. Furthermore, the NGF method, commonly used for MRD assessment as described previously, also enhanced CTC detection rates and highlighted its potential as a valuable prognostic biomarker [81,82,83]. Recently, the Spanish PETHEMA/GEM group presented the BloodFlow ultrasensitive approach, using NGF after immunomagnetic enrichment, enabling CTC detection below the 10^−6^ threshold associated with independent prognostic value for PFS [84]. Given the importance of a reproducible threshold, substantial efforts have been made to define broadly acceptable cut-off values with prognostic significance using high-sensitivity techniques (Table A1) [85,86,87,88,89,90]. Thus, advances in high-throughput methods have markedly improved the sensitivity and reproducibility of CTC detection, allowing their identification even in patients with low disease burden.

Elevated CTC levels constitute an independent prognostic marker in NDMM, correlating with reduced survival outcomes [81]. Recently presented data from the European CTC consortium revealed that NDMM patients with undetectable CTC by NGF (LOD of 2 × 10^−6^) enrolled in the GEM2012MENOS65, CLARIDEX, and GEM2017FIT clinical trials had significantly higher 5-year rates of PFS (80% versus 50%) and OS (92% versus 72%) compared to patients with detectable CTC. In multivariate analyses of PFS and OS, including transplant-eligibility and the R-ISS, undetectable CTC displayed independent prognostic value for PFS (hazard ratio (HR): 0.5; *p* = 0.005) and OS (HR: 0.4, *p* = 0.02) [91].

Beyond enumeration, the genomic characterization of CTC can offer prognostic information that is independent of, and complements, traditional staging systems and cytogenetic profiling. Elevated CTC counts are linked to complex genomic changes and the loss of key tumor suppressor genes, features often associated with high-risk disease [92]. High genetic concordance between CTC and BM PC supports its use as a reliable surrogate of the medullary clone, allowing minimally invasive monitoring of clonal evolution that overcomes limitations of BM sampling, including procedural invasiveness and spatial bias. The study by Mishima et al., analyzing CTC and BM tumor PC from 8 paired patients, showed that all clonal mutations found in BM were also present in CTC, and 99% of those identified in CTC were detected in BM tumor PC. These clonal somatic mutations include key driver genes such as *KRAS*, *NRAS*, and *BRAF* [93]. Whole-genome sequencing (WGS) analysis in a cohort of 24 patients using the Minimum Mutation Mapping sequencing (MinimuMM-seq) approach detected the same translocations and copy-number abnormalities in all paired BM and CTC samples [94]. Moreover, a study by the GEM/PETHEMA cooperative group comparing the genetic profile of BM PC, CTC, and extramedullary (EM) PC in 6 MM patients with EM involvement revealed that most targetable mutations were conserved across compartments. Notably, CTC showed the greatest mutational overlap with both BM and EM clones [95]. The prognostic relevance of CTC levels was also confirmed by the analysis of genomic and transcriptomic information of BM samples from 540 NDMM patients with available CTC in the CoMMpass dataset [96] and validated in an independent dataset of 135 NDMM patients [92]. Not only were higher CTC levels significantly associated with high-risk clinical features such as ISS, but also with complex genomic characteristics, including gain/amp1q, NSD2-related or MAF/MAFB-related translocations, chromothripsis, and APOBEC mutagenesis. Other non-invasive approaches, particularly cell-free DNA (cfDNA), which will be discussed later in this review, also show promise in monitoring clonal evolution.

Even after treatment, the presence of CTC has been identified as an independent prognostic marker. Lower numbers of CTC were associated with longer PFS, independent of therapy response as measured by CR status (*p* < 0.0001) or BM MRD status (*p* = 0.02) [97], defined as the presence of measurable residual cells in the BM detected by highly sensitive NGF beyond conventional response assessment. The persistence or presence of CTC might serve as a surrogate marker of BM MRD-positivity, since all treated patients who showed CTC after therapy were MRD-positive in paired BM samples. Patients with undetectable CTC who achieved MRD-negative CR showed unprecedented 5-year rates of PFS and OS (92% and 98%) [81].

#### Challenges to Clinical Implementation

CTC offers a minimally invasive approach for disease assessment. Nevertheless, its clinical utility is limited by several constraints. The extremely low frequency of CTC poses a fundamental challenge to their reliable detection and quantification. Achieving both a high yield and high purity when only a few CTC are present within a large background of normal cells is technically demanding. CTC counts are approximately 100-fold lower than in paired BM samples and, consequently, false-negative results are a major concern [98]. Limited analytical sensitivity, particularly in the context of MRD assessment, further restricts the broad clinical adoption of CTC-based assays. CTC detection and enrichment methods lack full standardization across platforms, making inter-laboratory comparisons difficult. High-sensitivity methods for CTC quantification, such as NGF, require fresh samples and fast processing time, introducing additional quality and logistical constraints, as well as an elevated degree of expertise to produce reliable results. This limits their availability to specialized centers and increases costs. Also, CTC detection in a relapse setting might be more difficult due to changes in phenotype after therapy exposure, complicating their detection using marker-based enrichment methods [99]. The low absolute CTC counts also make their isolation for subsequent use in downstream molecular studies challenging, either by immunomagnetic enrichment targeting CD138 or the CellSearch^®^ system, and interference from residual leukocyte contamination represents an added limitation [100,101]. Although CTC quantification has been implemented in clinical studies, sensitivity does not yet reach the gold standard level of BM-based MRD for all patients, and further validation and standardization are mandatory before it can serve as a stand-alone tool for MRD assessment [102]. Sequential CTC-based MRD monitoring has great potential to improve the impact of the existing risk stratification and response assessment models. One possible strategy could be to identify earlier signs of relapse, thus reducing the number of invasive BM aspirations. When integrated with complementary biomarkers such as cfDNA, CTC may better capture systemic tumor heterogeneity [100,103]. In this context, CTC reflects viable circulating cells actively released from BM, which may underrepresent focal or EM lesions that shed poorly into the blood and generate insufficient CTC for detection. Therefore, a multiparametric liquid biopsy strategy, combining CTC enumeration and genetics with cfDNA, could provide a broader coverage than either of them alone.

Recent consensus guidelines from the European Myeloma Network emphasize the growing role of CTC and other liquid biopsy approaches for prognostic stratification in NDMM, underscoring their potential integration into future clinical risk models, offering a complementary, patient-friendly approach [104].

### 2.3. Cell-Free DNA (cfDNA)

The analysis of cfDNA in PB has become a groundbreaking, noninvasive alternative to traditional techniques. It collects genetic material released from tumors throughout the body, offering a comprehensive, systemic view of disease extent, spatial diversity, and clonal changes [105]. A primary advantage of cfDNA is its ability to recapitulate the genomic landscape of the BM clone and provide a quantitative measure of tumor burden. Studies using ultra-low-pass whole-genome sequencing (ULP-WGS) and moderate-depth cfWGS have shown that the tumor fraction (TF) within cfDNA, often referred to as circulating tumor DNA (ctDNA), correlates significantly with established clinical biomarkers. For instance, ctDNA levels correlate strongly with β2-microglobulin, serum albumin, and the percentage of BM PCs plasma cells [106]. Similarly, in relapse/refractory MM (RRMM), cfDNA TF correlates with involved serum-free light chain (iFLC) levels [107]. cfDNA analysis can circumvent the limitations of hemodiluted or hypocellular BM samples. In a study involving 160 patients, 13.7% had uninformative BM samples because of low cellularity. However, in some of these cases, cfDNA detected copy number variations (CNVs), thereby preserving valuable genomic data and avoiding the need to repeat invasive procedures. Algorithms have been developed to create “plasma-only” classifiers trained on baseline cfDNA, which can stratify relapse risk and detect MRD even when diagnostic BM is unavailable or suboptimal [106].

The quantity of tumor-derived cfDNA is a potent and independent prognostic factor. A meta-analysis of 235 patients confirmed that higher levels of cfDNA/ctDNA are significantly associated with worse (HR 4.78) and OS (HR 3.06) [108]. Specific quantitative thresholds have been proposed to refine risk stratification. A ctDNA cutoff of >12% at diagnosis has been shown to distinguish patients with a poor prognosis, independent of standard high-risk factors such as R-ISS stage III and 1q amplification [108].

One of the most compelling applications of cfDNA is its ability to reflect the totality of tumor burden, with special clinical relevance in EM and paraskeletal (PS) disease, which are often missed by single-site BM aspiration [105,106]. Patients with high ctDNA levels at diagnosis exhibit a higher prevalence of metabolically active PS and EM lesions detected by positron emission tomography with computed tomography (PET/CT). In fact, higher levels of ctDNA were more likely to be associated with disease spread, especially beyond the BM. The integration of high ctDNA levels with PET/CT findings enables a multilayered risk assessment that captures disease dissemination more accurately than BM markers alone [106]. As such, the advantage of cfDNA in the context of spatial heterogeneity coverage is that it integrates information from all tumor sites simultaneously, making it inherently more representative in capturing EM and systemic disseminated disease than a single BM sample or even CTC counts, which depend on active tumor shedding into peripheral blood circulation. In oligo-secretory and traditionally non-measurable disease, cfDNA could be an alternative detection method when BM sampling is suboptimal and when MP is absent or below assay thresholds. Although dedicated studies are absent, this approach could also be useful in MM with renal impairment, as cfDNA is unaffected by renal clearance, though it might increase background cfDNA from tissue injury, elevating total cfDNA and requiring careful tumor fraction quantification.

While cfDNA holds great promise, its sensitivity relative to other liquid biopsy components, such as CTC, varies with the analytical method used. In a direct comparison using NGS of Ig genes (NGS-IG), enriched CTC showed superior sensitivity, detecting tumor DNA in 100% of active disease samples, compared with 76% for cfDNA. Specifically, for MRD detection in remission, enriched CTC identified residual disease in 50% of BM-MRD-positive patients, whereas cfDNA showed lower clonal Ig sequence detection rates [109]. This suggests that for Ig-targeted sequencing, cellular enrichment may be necessary to maximize sensitivity. However, cfDNA appears superior for other genomic applications. For non-invasive DNA methylation profiling, cfDNA showed significantly higher concordance with BM-DNA (78.2%) than with CTC-DNA (53.3%) or PB mononuclear cells [109]. This indicates that cfDNA is a robust biomarker for epigenetic profiling, capable of identifying hypermethylation in tumor suppressor genes and resistance-associated regions. Additionally, DNA-based, phenotypic changes do not directly impair detection. Therefore, the choice between cfDNA and CTC is not either/or but application-dependent, on whether the clinical goal is Ig-based MRD tracking, where CTC shows superior sensitivity, or broader genomic/epigenetic characterization. Both have complementary roles in capturing the special heterogeneity of MM.

The criteria established by the IMWG are predominantly based on serological markers, which can exhibit a delay in reflecting underlying biological tumor activity. cfDNA analysis has demonstrated the capacity to refine these response categories. Among patients classified as having Stable Disease (SD) or Partial Response (PR) by IMWG criteria, cfDNA status could further segregate them into distinct prognostic groups. For example, those with residual cfDNA positivity had significantly shorter PFS than those who were cfDNA-negative, despite identical clinical response [107]. Additionally, in the post-transplant setting, cfDNA positivity at day +100 has been associated with early recurrence, even in cases where standard MRD assessment was negative, suggesting it may capture unique risk mechanisms or extramedullary reservoirs [110].

#### Challenges to Clinical Implementation

Cell-free DNA analysis shows promise in MM as a minimally invasive tool. As plasma can be frozen, it is more flexible and has fewer logistical constraints. However, numerous important limitations hinder its widespread clinical application. One key challenge is its lower sensitivity compared to BM-based assays, particularly in the MRD setting. This is partly due to the short fragment length of cfDNA, which makes amplicon generation for Ig-based NGS more technically challenging and may result in lower tumor detection rates (e.g., 76%) compared with enriched CTC approaches, which detect 100%. This limits cfDNA’s usefulness in the identification of the dominant clone, as “pseudoclonal” sequences may arise from limited template availability [111]. Biological factors also influence cfDNA performance. Patients with focal disease or low-tumor DNA shedding confined to the BM may have negative blood-based results despite persistent disease in the BM, leading to false-negative findings. Additionally, total cfDNA levels can be influenced by non-tumor-related factors, such as inflammation, infection, or tissue injury, making it essential to assess the tumor-derived fraction rather than total cfDNA concentration alone [105]. From a technical and operational perspective, several barriers remain, including the lack of standardized protocols for sample processing, sequencing methods, and target gene panels. The genetic heterogeneity of MM also requires deep sequencing infrastructures and bioinformatics pipelines, which increase costs and add complexity. Furthermore, large prospective multicenter studies are still needed to validate prognostic thresholds, demonstrate clinical utility, and support regulatory approval and routine clinical implementation [105,111]. Overall, while cfDNA represents a promising tool for disease monitoring and molecular profiling, further standardization, technical optimization, and clinical validation are required before it can be fully integrated into standard MM management.

### 2.4. Cell-Free RNA (cfRNA)

The analysis of cell-free RNA (cfRNA), also referred to as extracellular RNA (exRNA), has become an important and complementary method for non-invasively studying the global transcriptome. Unlike cfDNA, which mainly originates from cell death, circulating RNAs are produced through both cell death and active vesicle secretion, offering a dynamic view of the tumor’s functional state [112,113]. A whole-transcriptome study demonstrated that approximately 45% of plasma-derived cfRNA genes in MM patients are protein-coding, with a vast majority of identified genes showing high sequence coverage [113]. This stability enables effective recapitulation of BM transcriptomic features. For instance, integrated sequencing of plasma-derived cfDNA and cfRNA has shown a median concordance of 85.7% with matched BM samples, validating the utility of this method for molecular characterization at diagnosis [114]. The profiling of cfRNA has identified genes that are significantly differentially expressed, distinguishing MM patients from healthy controls and separating disease stages. *GOLGA8O* and *TRAK2* have been identified as common dysregulated genes across both NDMM and RRMM patients. *TRAK2* downregulation is particularly notable given its role in endosome-to-lysosome trafficking, a process relevant to exosome biogenesis. Specific transcriptomic signatures have been identified for different disease phases. *MYOD1* and *UBB* are upregulated in NDMM, while IER3, a gene with anti-apoptotic functions in myeloma cells, is upregulated in RRMM. Furthermore, cfRNA analysis facilitates the detection of specific variants, such as non-synonymous single-nucleotide polymorphisms (SNPs) in mucin family genes (MUC3A, MUC5AC), which are more common in relapsed patients [113]. Beyond diagnosis, cfRNA offers unique insights into therapeutic monitoring and mechanisms of drug resistance. Baseline cf-mRNA levels of *CRBN* and *IKZF1/3* have been associated with the risk of early progression and can serve as biomarkers of response to immunomodulatory drugs such as lenalidomide [112].

#### Challenges to Clinical Implementation

Despite the potential of cfRNA to provide a comprehensive and dynamic view of the MM transcriptome, several limitations currently restrict its clinical implementation. One of the main challenges is the intrinsic instability of RNA molecules. cfRNA is highly prone to degradation by circulating RNases, and its low abundance in plasma makes extraction and analysis technically challenging. Although encapsulation within extracellular vesicles can protect RNA from degradation, pre-analytical variables such as sample collection, processing time, storage conditions, and RNA isolation methods can significantly affect cfRNA quality and reproducibility, highlighting the need for standardized protocols. Another important limitation is the requirement for high sequencing depth to obtain reliable transcriptomic data, which increases costs and computational complexity. While whole-transcriptome sequencing yields broad information on gene expression profiles, it may suffer from uneven coverage and low sensitivity for specific low-expression or clinically relevant genes. Hotspot mutations in genes such as *KRAS*, *NRAS*, and *TP53* can be missed by RNA sequencing even when present in the tumor cells. This means that cfRNA analysis cannot currently replace DNA-based sequencing for full genomic characterization and must be used as a complementary approach [113]. Additionally, the biological interpretation of cfRNA results remains challenging, as circulating RNA may originate not only from tumor cells but also from normal hematopoietic and non-hematopoietic cells, inflammatory processes, or tissue damage, potentially confounding tumor-specific signals. There is also currently very limited clinical validation of cfRNA biomarkers in large prospective MM cohorts, as well as no standardized thresholds or clinically approved assays available. Regulatory approval, cost, and the need for specialized bioinformatic analysis further limit widespread adoption. In less common clinical settings, such as oligo-secretory and traditionally non-measurable disease, MM with renal impairment, and EM disease, the use of cfRNA is still highly exploratory, and no dedicated studies or validation data are available.

The optimal application scenarios, available detection thresholds, and key limitations of each non-invasive approach across disease stages are summarized in Table 3. Post-ASCT was included as it remains a central standard-of-care option for eligible patients. Although outside the primary scope of this review, the inclusion of the precursor stages MGUS and SMM acknowledges the gap in assessment during these early phases that remain.

## 3. Molecular Markers: Deepening the Genomic Picture

MM displays marked genomic complexity and dynamic clonal heterogeneity, evolving over time and under therapeutic pressure [115,116]. Although conventional cytogenetics and MRD assessment provide important prognostic information, they capture only selected dimensions of disease biology [117]. Accumulating genomic evidence demonstrates that additional molecular alterations beyond baseline cytogenetic risk and MRD contribute to disease progression, therapy resistance, and relapse. This has driven increasing interest in incorporating emerging molecular markers into diagnostic and monitoring approaches to better reflect the underlying biological diversity and evolutionary behavior of MM [118]. MM comprises multiple biologically distinct genomic subgroups defined by characteristic combinations of driver mutations, structural variants, copy-number changes, and mutational processes, each associated with different clinical trajectories [119].

Expert consensus further emphasizes that, despite major therapeutic progress, a biologically defined subset of high-risk MM continues to have inferior outcomes, underscoring the need for biomarkers capable of capturing aggressive disease biology [120]. Common secondary genetic events acquired during the course of disease include copy-number abnormalities such as loss of chromosomal regions 17p13 and 1p32 and the gain of the long arm of chromosome 1 (1q gain), associated with shorter survival [121,122,123]. Chromosome 1q amplifications (amp1q, more than three copies) may induce an even worse prognosis than a gain (three copies) [124]. Mutations affecting pathways related to DNA repair, cell-cycle regulation, and MAPK signaling (*KRAS*, *NRAS*, *BRAF*), and *TP53* alterations have also been repeatedly associated with clonal expansion, relapse, and progression [125,126]. MM clonal evolution can proceed through linear progression, branching evolution, or shifting dominance of competing subclones, particularly under therapeutic pressure [116,127].

While fluorescence in situ hybridization (FISH)-based cytogenetic profiling remains the clinical standard for baseline risk stratification, its limited resolution and locus-restricted scope constrain its ability to capture the full spectrum of genomic complexity [128]. Comprehensive genomic studies have shown that MM harbors a wide range of somatic mutations, structural variants, and copy-number alterations that exceed the detection capacity of FISH, supporting the complementary use of NGS for a more complete characterization of genomic risk [129]. Copy-number variations, genomic alterations defined by gains or losses of chromosomal segments, represent key contributors to biological risk in MM. NGS-based CNVs assessment has repeatedly shown superior analytical resolution and sensitivity compared with FISH, particularly for clinically relevant lesions such as gain(1q) [130]. Molecular analyses have also demonstrated that alterations involving *TP53*, biallelic inactivation of tumor-suppressor genes, chromothripsis, and other forms of complex genomic instability represent high-risk abnormalities that can co-occur within the same clone [116,120]. They are strongly associated with more aggressive clinical behavior and inferior outcomes, supporting the biological concept of “multi-hit” MM [117,120]. In this context, 20 recurrent genomic alterations with independent prognostic significance have been identified, enabling a more precise characterization of the molecular architecture of risk in multiple myeloma [117].

Therapeutic interventions exert strong selective pressure on the myeloma clone, progressively reshaping the genomic landscape. Relapses are frequently characterized by the expansion of pre-existing resistant subclones or the emergence of newly acquired alterations that confer survival advantages. Longitudinal sequencing analyses confirm that clonal composition at relapse often differs substantially from that observed at diagnosis, supporting the need to re-evaluate molecular risk at key clinical timepoints. Recurrent enrichment of MAPK pathway mutations, gain of 1q, and loss of 17p illustrates important mechanisms through which molecular evolution contributes to treatment resistance [115,116,131]. Evidence from whole-genome and multi-region sequencing studies indicates that specific genomic events, such as 1q amplification and *TP53* inactivation, tend to arise as later steps in disease evolution and are frequently associated with relapse or treatment-refractory disease. These recurrent patterns do not define a universal linear trajectory but suggest that specific secondary aberrations repeatedly emerge under therapeutic selective pressure, contributing to clonal diversification and disease recurrence [132,133].

### Challenges to Clinical Implementation

Although cytogenetic abnormalities detected by FISH remain the only genomic markers formally recommended for routine risk stratification in MM, recent genomic studies have identified several molecular alterations with potential prognostic relevance (Table 4). Some alterations, such as BRAF V600E, although rare, may also have therapeutic relevance in selected relapsed or refractory cases due to potential sensitivity to targeted inhibitors [134]. Additional emerging molecular features include mutational signatures such as APOBEC and complex forms of genomic instability such as chromothripsis [115,116,124]. However, despite their biological and clinical interest, none of these molecular biomarkers are currently validated or recommended for routine clinical decision-making to define high-risk MM, and are not included in the International Myeloma Society-IMWG consensus risk stratification criteria [15]. Therefore, their use remains limited to research or specialized centers, where they may provide additional insight into disease aggressiveness or clonal evolution, particularly in cases where cytogenetic findings alone do not fully explain clinical behavior [131,132].

Several limitations still hinder the integration of molecular profiling into routine clinical practice. One major limitation is the lack of standardization across sequencing platforms, gene panels, bioinformatic pipelines, and reporting criteria. This complicates the comparison between studies and clinical implementation. In addition, the prognostic impact of many molecular alterations is influenced by clonal context, co-occurring abnormalities, tumor burden, and treatment exposure, making interpretation more complex than traditional cytogenetic markers. Another important limitation is that most available evidence comes from retrospective analyses or relatively small cohorts, and large prospective clinical trials demonstrating clear clinical benefit from molecularly guided risk stratification or treatment selection are still limited. Cost, infrastructure requirements, and access to high-throughput sequencing technologies also represent important barriers, particularly outside specialized centers. Furthermore, tumor heterogeneity and clonal evolution mean that a single baseline molecular assessment may not fully reflect disease biology over time, highlighting the need for longitudinal molecular monitoring, which is not yet standardized or routinely implemented.

Complementary guidance from the European Myeloma Network NGS consensus emphasizes the expanding role of NGS in baseline risk assessment and molecular characterization, but similarly concludes that most molecular alterations beyond established cytogenetic markers currently lack sufficient evidence to guide routine clinical decision-making [104]. Nevertheless, early precision-medicine studies in relapsed and refractory MM suggest that integrated molecular and functional profiling may help identify actionable alterations and guide personalized therapy in selected patients. In a real-world cohort, integrated profiling combining NGS with functional protein-expression markers identified genomic alterations such as *KRAS*, *NRAS*, *BRAF*, *TP53*, and drug-sensitivity markers such as TOP1, TOP2A, TUBB3, ERCC1, MGMT, and RRM1. Importantly, biomarker-guided therapy selection led to clinically meaningful benefit in a subset of patients, including prolonged PFS compared to the prior treatment line [135]. These findings support the future clinical integration of molecular profiling, although further prospective validation, standardization, cost reduction, and guideline development are required before widespread implementation.

## 4. Artificial Intelligence (AI): Transforming Diagnostic and Therapeutic Decisions

Artificial intelligence (AI) and machine learning (ML) are reshaping laboratory hematology by addressing the most persistent challenges: the subjectivity of morphological assessment, the complexity of multi-modal data integration, and the labor-intensive nature of manual diagnosis [136]. AI and ML are being explored as tools to support interpretation of heterogeneous data over time, including laboratory results, BM assessments, imaging studies, and real-world clinical parameters, complementing established laboratory and clinical workflows rather than replacing them [137,138]. AI-based approaches have also been investigated for early MM detection using routine laboratory data, enabling pre-diagnostic identification before overt clinical presentation [139].

In morphological analysis, AI-driven digital pathology is overcoming inter-observer variability and manual fatigue [136]. AI and ML are mainly applied to improve the interpretation, standardization, and reproducibility of data from technically demanding assays. At diagnosis, ML algorithms can predict MM directly from raw MFC data [140]. In MRD assessment by NGF, the standardized antibody panels, instrument settings, and analysis strategies developed within the EuroFlow consortium have enabled reproducible high-dimensional datasets. This framework supports computational and ML-based approaches to assist with gating strategies, which refer to the process of defining cell populations based on marker expression in flow cytometry and are traditionally operator-dependent and subject to variability, reduce inter-operator variability, and improve detection of rare abnormal PCs [139,141,142]. Similarly, ML methods are being explored in NGS workflows to optimize data processing and facilitate the interpretation of complex molecular profiles, while in MS-based assays, AI is mainly applied to data analytics and workflow support, including pattern recognition within complex spectra and longitudinal tracking of patient-specific signals. Across laboratory modalities, AI should be viewed as an enabling technology that improves analytical consistency and supports longitudinal interpretation, rather than functioning as an autonomous diagnostic system [143,144,145].

One of the most relevant clinical applications of AI is longitudinal disease monitoring. ML models have been developed to analyze time-series data from serial laboratory measurements, BM evaluations, and treatment timelines to identify early patterns associated with disease progression or relapse. This approach is particularly relevant in MRD-driven monitoring, where disease burden may fluctuate below the detection limits of individual assays for prolonged periods [146]. Predicting MRD negativity is also important for treatment personalization. An ML model integrating tumor burden, cytogenetics (del(17p), t(4;14)), and immune biomarkers was able to predict MRD outcomes in up to 72% of NDMM patients [147]. Analyses of large clinical trial datasets and real-world cohorts demonstrated the feasibility of ML models to predict disease progression using longitudinal laboratory and clinical parameters, supporting the potential role of AI in dynamic risk stratification [138]. A model developed by the Spanish Myeloma Group reclassified R2-ISS patients into distinct risk groups with predictive independent value of induction or transplant status [148]. The Individualized Risk Model Myeloma (IRMMa) further integrated demographic, clinical, and genomic data using a neural Cox non-proportional hazards architecture. Trained on nearly 2000 patients, IRMMa achieved superior C-indices for event-free and OS than R2-ISS and maintained performance even with missing data or absent genomic information [117].

AI models are also being explored to optimize therapeutic sequencing. The IAC-50 model evaluated first-line regimens and found that only 41% of patients historically received the “optimal” regimen. The model can inform the choices between agents (e.g., bortezomib versus carfilzomib) and specific triple combinations while retaining predictive value across age groups and transplant eligibility [149,150]. Other models have used baseline data to guide therapy selection [151]. Although these approaches highlight the potential of AI to support individualized treatment strategies, current evidence remains largely investigational, with limited prospective validation and no established role in routine clinical decision-making. Accordingly, AI should be considered a complementary tool that may inform, but not replace, clinician-led therapeutic decisions [138,152,153].

### Challenges to Clinical Implementation

Despite encouraging developments, several limitations currently constrain the clinical implementation of AI in MM monitoring. These include the need for robust external validation, generalizability across institutions, transparency and interpretability of AI models, as some approaches may operate as “black boxes”, meaning that their decision-making processes are not easily interpretable, which may limit clinical trust. In contrast, explainable AI approaches aim to improve transparency and facilitate clinical adoption, and integration into existing laboratory and clinical workflows. Currently, training data are predominantly of NDMM, to define risk models validated in retrospective cohorts, with limited precursor-stage, and prospective post-transplant data. High heterogeneity in the RRMM setting also limits model robustness, and training cohorts for rare resistance patterns will be needed to assist in relapse prediction, drug sensitivity and resistance modelling. Data quality, bias, and interoperability remain critical challenges, particularly for multimodal approaches combining laboratory, imaging, and real-world data. Addressing these issues will be essential to ensure that AI-based tools provide reliable and clinically meaningful support in MM monitoring [154].

## 5. Future Directions: The Multi-Omics Era in the Clinical Setting

The translation of laboratory innovations into clinical practice should be guided by clearly defined clinical needs. In MM, key priorities include accurate MRD monitoring, improved identification of patients at high-risk of progression, and strategies to overcome therapeutic resistance. Achieving these goals requires standardized analytical pipelines and quality-controlled workflows to allow result comparison and clinical interpretation. Despite the significant advances in laboratory technologies, as discussed previously, several limitations still hinder their widespread clinical implementation. Many of these techniques are associated with high costs, a need for specialized equipment, and highly trained personnel, which limits their availability to specialized centers. Another important limitation is the relatively limited validation of some of these methods in large prospective clinical trials, and as such, their prognostic and clinical utility is still being defined. Regulatory approval and integration into clinical guidelines are also ongoing challenges for several emerging methodologies. Furthermore, logistical issues such as sample processing requirements, turnaround time, and data analysis complexity are particularly relevant for high-throughput and single-cell technologies that generate large-scale datasets to be analyzed.

While some technologies are currently validated as prognostic biomarkers, not all have been prospectively tested as decision-making tools. As such, further prospective interventional data linking each technology use to clinical endpoint improvement is still lacking and requires future studies. Nevertheless, these innovative laboratory approaches hold great promise for improving diagnosis, risk stratification, and disease monitoring if the limitations to their clinical implementation are tackled. Particularly in less common disease subtypes, where the clinical need for highly sensitive and less spatially dependent assessment is greater, performance evaluation and validation studies are imperative. Minimally invasive liquid biopsy approaches represent an important complementary strategy. Analysis of circulating tumor components such as CTC, cfDNA, cfRNA, or MP in PB may overcome limitations of BM-based assessments. Given spatial heterogeneity and patchy BM infiltration, these may provide a more representative evaluation of disease burden while enabling serial sampling and longitudinal monitoring with minimal patient discomfort. The role of MS in MM is expected to expand as response-adapted treatment strategies are increasingly adopted. Integration of serum-based MS with BM MRD assessment and advanced imaging may enable more comprehensive disease evaluation, supporting personalized monitoring and risk stratification, particularly when repeated BM sampling is impractical. Additional advances may arise from single-cell-based MRD assessment, where combining NGF for CTC enrichment with scDNA-seq may enhance detection sensitivity and disease surveillance. In RRMM, these technologies also provide insights into resistance mechanisms and support biomarker discovery for therapeutic development. Finally, AI-driven analytical pipelines and database-assisted annotation may further improve data interpretation and standardization. Generative AI tools are increasingly used to process unstructured clinical data, supporting clinical decision-making and reducing clinician workload, although rigorous prospective validation will be required to ensure meaningful clinical benefit.

## 6. Conclusions

Traditionally, the diagnosis and monitoring of MM have centered on established biomarkers, including β2-microglobulin, albumin, and SPE, later supplemented by advanced imaging modalities, cytogenetic analysis, and serum light chain quantification. Although these methods have substantially enhanced disease evaluation, their inherent limitations underscore the necessity for more sensitive biomarker discovery. Achieving precision medicine in MM requires the integration of robust high-throughput technologies with advanced bioinformatics platforms capable of managing complex clinical, biological, genetic, and immunological datasets. It is anticipated that, over time, laboratory advancements will be incorporated into the standard of care, facilitating a more personalized approach to MM management.

## Figures and Tables

**Table 1:** Diagnostic criteria for MM and the corresponding assessment methods (adapted from [3]).

Domain	Specific Criteria	Threshold/Definition	Primary Method(s)/Technique(s)
MP	Serum MP	Any detectable level (≥3 gm/dL)	SPE; sIFE; quantitative Ig
	Urinary MP	Any detectable level (≥500 mg/24 h)	24-h UPEP; uIFE
	Serum FLC	Abnormal κ/λ ratio	Serum FLC assay
Diagnosis of active MM (plus 1 or more MDE)	BM clonal PCs	≥10% clonal PCs in BM aspirate/biopsy	BM aspiration and trephine BP; morphologic assessment; MFC; IHC
	BP-proven plasmacytoma	Extramedullary or bone plasmacytoma	Core needle or surgical BP; histopathology with IHC
CRAB features	Hypercalcemia (C)	Serum calcium > 0.25 mmol/L above upper limit of normal or >2.75 mmol/L (>11 mg/dL)	Calcium levels (colorimetric spectrophotometry or ion-selective electrode methods)
	Renal insufficiency (R)	Creatinine clearance < 40 mL/min or serum creatinine > 177 µmol/L (>2 mg/dL)	Serum creatinine (enzymatic assays); eGFR; creatinine clearance
	Anemia (A)	Hemoglobin < 10 g/dL or >2 g/dL below lower limit of normal	CBC (automated hematology analyzer)
	Bone disease (B)	≥1 osteolytic lesion	Whole-body low-dose CT (preferred); PET/CT; MRI; skeletal survey (radiographs, historical)
SLiM features	BM clonal PCs (S)	≥60% clonal PC	BM BP with morphologic count ± MFC
	Serum involved/uninvolved FLC ratio (Li)	≥100 (with involved FLC ≥ 100 mg/L)	Serum FLC assay
	MRI focal lesions (M)	>1 focal lesion ≥ 5 mm	Whole-body MRI or spine/pelvic MRI
	Urinary MP	Any detectable level (≥200 mg/day)	24-h UPEP; uIFE
	Serum FLC	Abnormal κ/λ ratio	Serum FLC assay

MM: multiple myeloma; MP: monoclonal protein; FLC: free-light chain; SPE: serum protein electrophoresis; sIFE: serum immunofixation electrophoresis; Ig: immunoglobulins; UPEP: urine protein electrophoresis; uIFE: urine immunofixation electrophoresis; MDE: myeloma-defining events; PC: plasma cell; BM: bone marrow; BP: biopsy; MFC: multicolor flow cytometry; IHC: immunohistochemistry; CBC: complete blood count; eGFR: estimated glomerular filtration rate; CT: computed tomography; PET/CT: positron emission tomography with CT; MRI: magnetic resonance imaging.

**Table 2:** Selected clinical trials evaluating MRD-guided strategies in MM.

Trial	Treatment	MRD Strategy and Implication
PERSEUS	Dara-VRd → ASCT → Dara-R maintenance	Evaluate maintenance cessation after ≥24 months in sustained MRD-negative patients
DRAMMATIC	Maintenance therapy	Evaluate MRD-adapted maintenance duration
MASTER	Dara-KRd → ASCT → consolidation	Feasibility of MRD-guided therapy cessation after sustained MRD < 10^−5^ (MRD-SURE phase— surveillance only)
MASTER-2	Dara-VRd ± ASCT	Feasibility of adaptive MRD strategy: ASCT deferral if MRD-negative Intensify if MRD-positive
MIDAS MRD2STOP	MRD-guided consolidation/stop	Assess safe discontinuation of maintenance in deep MRD responses (<10^−7^)
FORTE; MAIA; ALCYONE; POLLUX; CASTOR	Dara-based regimens	Evaluate longitudinal MRD Dynamics to predict better outcome
DREAMM-7 DREAMM-8	Belantamab mafodotin regimens	Confirm prognostic values of MRD assessment
TOURMALINE-MM3 TOURMALINE-MM4 AURIGA	Ixazomib or Dara maintenance	Associate depth and persistence of MRD negativity with outcome

MRD: measurable residual disease; MM: multiple myeloma; Dara: daratumumab; V: bortezomib; R: lenalidomide; d: dexamethasone; ASCT: autologous stem cell transplantation; K: carfilzomib.

**Table 3:** Summary of the optimal application scenario, available detection threshold, and key limitations of each non-invasive approach across disease stages.

**MS**				
**Parameter**	**MGUS/SMM**	**NDMM**	**Post-ASCT**	**RRMM**
Application	Detection of MP below SPE/sIFE thresholds; identifies clonal proteins in MGUS not detectable by conventional methods; may improve risk stratification	Baseline MP quantification and isotype characterization; superior sensitivity vs. SPE/sIFE; particularly valuable in oligosecretory disease	Complementary to BM for MRD detection; detects residual MP in CR/sCR; provides earlier relapse signal than sIFE; MS MRD negativity as stringent remission criterion	Sensitive relapse detection before clinical progression; identifies re-emerging MP in apparent CR; tracks isotype switching, Mass-Fix positivity independently predicts PFS/OS
Threshold	Mass-Fix sensitivity ~1–5 mg/L; superior to sIFE for low-level MP	Mass-Fix detects monoclonal Ig at <1 g/dL; MS MRD negativity combined with NGF/NGS associated with deeper remission	MS MRD negativity plus NGF/NGS MRD negativity associated with optimal post-transplant remission category	Mass-Fix positivity post-treatment is an independent PFS/OS predictor; earlier signal than sIFE
Limitations	Oligosecretory/traditionally non-measurable disease undetectable; cannot replace immunophenotyping or BM assessment	Oligosecretory/traditionally non-measurable disease undetectable; not yet universally standardized across laboratories	Integration with NGF/NGS MRD not yet fully standardized; Oligosecretory/traditionally non-measurable disease undetectable excluded	Requires high-sensitivity platform; small MP at early relapse may still be near detection limit
**CTC**				
**Parameter**	**MGUS/SMM**	**NDMM**	**Post-ASCT**	**RRMM**
Application	Exploratory risk stratification in high-risk SMM; CTC presence may predict progression independently of BM burden	Independent prognostic marker; detectable in 70–87% by NGF; undetectable CTC associated with 5-year PFS 80% vs. 50% and OS 92% vs. 72%	CTC persistence post-treatment may be a surrogate for BM MRD positivity; undetectable CTC plus MRD with negative CR: 5-year PFS 92%, OS 98%	Surrogate for BM MRD status; clonal evolution monitoring; genomic characterization of relapse clone (*KRAS*, *NRAS*, *BRAF*)
Threshold	Not yet validated for precursor stages	LOD ~2 × 10^−6^ (NGF/BloodFlow)	Detectable vs. undetectable (*p* = 0.02 vs. BM MRD)	Detectable vs. undetectable; lower counts associated with longer PFS independent of CR status (*p* < 0.0001)
Limitations	Very low CTC counts; high false-negative rate; no guideline recommendation	Fresh sample required; ~100× lower count than BM; expertise-dependent	Sensitivity inferior to BM MRD for all patients; not yet a stand-alone MRD tool	Therapy-induced phenotypic shift complicates marker-based selection; standardization lacking
**cfDNA**				
**Parameter**	**MGUS/SMM**	**NDMM**	**Post-ASCT**	**RRMM**
Application	Non-invasive detection of CNVs and early genomic instability in SMM; plasma-only risk classifiers under development	Tumor burden quantification; EM/PS disease detection correlated with PET/CT findings; plasma-only classifiers when BM unavailable or hypocellular	cfDNA positivity at day +100 predicts early relapse even when standard MRD is negative; captures extramedullary reservoirs missed by BM sampling	Refines IMWG response categories; cfDNA-positive SD/PR patients have significantly shorter PFS than cfDNA-negative counterparts; resistance mutation detection
Threshold	Tumor fraction not validated at precursor stage; low shedding limits utility	ctDNA >12% associated with poor prognosis independent of R-ISS; lower PFS/OS	Positivity at day +100 is early relapse predictor beyond standard MRD	cfDNA positivity/negativity within same IMWG category as independent PFS predictor; iFLC correlation
Limitations	Minimal tumor DNA shed in MGUS/SMM; high false-negative rate	Lower sensitivity than enriched CTC for Ig-MRD (76% vs. 100%); inflammation confounds total cfDNA	Post-ASCT tissue injury elevates total cfDNA; tumor fraction must be assessed separately	Focal BM disease may yield false negatives; deep sequencing costs; no standardized panels
**cfRNA**				
**Parameter**	**MGUS/SMM**	**NDMM**	**Post-ASCT**	**RRMM**
Application	Exploratory transcriptomic profiling of precursor clones	Gene expression profiling without BM aspirate	Exploratory relapse prediction signatures	Tracking transcriptomic changes associated with acquired resistance; complement to cfDNA
Threshold	Not established	No consensus panel	Not established	Not established
Limitations	Highly unstable analyte; no validated markers or clinical thresholds in precursor disease	RNA instability; pre-analytical sensitivity; significant inter-study variability; not clinically validated	No prospective post-transplant validation; largely exploratory	scRNA-seq from circulating material logistically demanding; limited to research settings

MGUS: monoclonal gammopathy of undetermined significance; SMM: smoldering multiple myeloma; MM: multiple myeloma; ASCT: autologous stem cell transplant; RRMM: relapsed/refractory MM; MS: mass spectrometry; MP: monoclonal protein; SPE: serum protein electrophoresis; sIFE: serum immunofixation electrophoresis; BM: bone marrow; MRD: Measurable Residual Disease; CR: complete remission, sCR: stringent CR; PFS: progression-free survival; OS: overall survival; Ig: immunoglobulins; NGF: next-generation flow; NGS: next-generation sequencing; CTC: circulating tumor cells; LOD: limit of detection; cfDNA: cell free DNA; CNVs: copy-number variations; EM: extramedullary; PS: paraskeletal; PET/CT: positron emission tomography with computed tomography; IMWG: International Myeloma Working Group; SD: stable disease; PR: partial response; R-ISS: Revised International Staging System; iFLC: involved free-light chain; cfRNA: cell free RNA; scRNA-seq: single cell RNA sequencing.

**Table 4:** Clinical relevance and evidence level of emerging molecular biomarkers in MM.

Molecular Marker	Current Recommendations	Observations
*TP53* mutation	Consider in extended NGS panels	High-risk biology Complements del(17p) but not a standalone HR criterion
RAS/MAPK mutations (*KRAS*, *NRAS*, *BRAF*)	Optional in NGS profiling	Associated with progression and resistance Not validated for HR stratification
DIS3, FAM46C, CYLD mutations	Limited routine use (mostly research-supportive)	Associated with RNA processing/NF-κB pathways Variable prognostic value
Mutational burden	Not recommended for routine	Correlates with genomic instability; lacks validated cutoffs
APOBEC mutational signatures	Research only	Strongly associated with aggressive/relapsing MM
Whole-genome instability patterns (chromothripsis, templated insertions)	Investigational but promising	Powerful predictors of poor outcome Requires WGS
Gene expression profiling (GEP70, SKY92)	Promising but not standardized	Identifies molecular HR Not included in HR MM consensus
Single-cell molecular profiling (scRNA-seq, scDNA-seq)	Research only	Refine clonal hierarchy and evolution
Multi-omic risk scores (integrating genomics, transcriptomics, epigenomics)	Investigational	Likely use for precision stratification in the future
ctDNA	Under validation for future clinical use	Captures whole-body tumor genomics Correlates with MRD
cfDNA fragmentation and/or methylation patterns	Emerging	Traces clonal evolution Early relapse detection
PB NGS for genomic profiling	Under development	Potential alternative to BM sequencing

MM: Multiple Myeloma; NGS: Next-Generation Sequencing; HR: High-Risk; APOBEC: Apolipoprotein B mRNA Editing Catalytic Polypeptide-like; MRD: Measurable Residual Disease; PB: Peripheral Blood; BM: Bone Marrow.

## Data Availability

No new data were created or analyzed in this study.

## Abbreviations

The following abbreviations are used in this manuscript:

MM	Multiple myeloma
PCs	Plasma cells
BM	Bone marrow
MGUS	Monoclonal gammopathy of undetermined significance
SMM	Smoldering MM
IMWG	International Myeloma Working Group
MDE	Myeloma-defining events
IGH	Immunoglobulin heavy chain
ISS	International Staging System
R-ISS	Revised ISS
R2-ISS	2nd Revised ISS
CGS	Consensus Genomic Staging
CR	Complete response
sCR	Stringent CR
MRD	Measurable residual disease
PFS	Progression-free survival
OS	Overall survival
FDA	Food and Drug Administration
NDMM	Newly diagnosed MM
NGF	Next Generation Flow
NGS	Next Generation Sequencing
LLOD	Lower limit of detection
LLOQ	Lower limit of quantification
DNA	Deoxyribonucleic acid
MS	Mass spectrometry
SPE	Serum protein electrophoresis
IFE	Immunofixation electrophoresis
sFLC	Serum free light chain
MP	Monoclonal protein
QIP	Quantitative immunoprecipitation
CTC	Circulating tumor cells
PB	Peripheral blood
MFC	Multiparametric flow cytometry
HR	Hazard ratio
EM	Extramedullary
RR	Relapsed/refractory
cfDNA	Cell-free DNA
scRNA	Single-cell RNA
ULP	Ultra-low-pass
WGS	Whole-genome sequencing
TF	Tumor fraction
ctDNA	Circulating tumor DNA
RRMM	Relapsed/refractory MM
iFLC	Involved free light chain
CNVs	Copy number variations
PS	Paraskeletal
PET/CT	Positron Emission Tomography-Computed Tomography
Ig	Immunoglobulin
SD	Stable disease
PR	Partial response
cfRNA	Cell-free RNA
exRNA	Extracellular RNA
SNPs	Single-nucleotide polymorphisms
FISH	Fluorescence in situ hybridization
AI	Artificial intelligence
ML	Machine learning
IRMMa	Individualized Risk Model Myeloma

## Appendix A

**Table A1:** Reported MRD cut-off thresholds determined by MFC or NGF across different studies.

Study	Method	Proposed MRD Cut-Off
Garcés et al. [85]	NGF	≥0.01%
Tembhare et al. [86]	NGF	≥0.01%
Kastritis et al. [87]	NGF	≥0.0014%
Bae et al. [88]	5-color MFC	≥0.02%
Kostopoulos et al. [89]	8-color MFC	≥0.02%
Bertamini et al. [90]	8-color MFC	≥0.07%

MRD: Measurable residual disease; MFC: Multicolor flow cytometry; NGF: Next-generation flow.
